# Blood ammonia concentration measurement – effects of sampling site and cirrhosis during induced hyperammonaemia

**DOI:** 10.1007/s11011-024-01442-4

**Published:** 2024-11-20

**Authors:** Lars Djernes, Hendrik Vilstrup, Peter Ott, Peter Lykke Eriksen

**Affiliations:** 1 Department of Hepatology and Gastroenterology, Aarhus University Hospital, Aarhus, Denmark; 2 Department of Anaesthesiology and Intensive Care, Viborg Regional Hospital, Viborg, Denmark; 3 Department of Anaesthesiology and Intensive Care, Aarhus University Hospital, Aarhus, Denmark; 4 Department of Clinical Medicine, Aarhus University, Aarhus, Denmark

**Keywords:** Ammonia, Sampling site, Arterial-venous concentration differences, Cirrhosis, Upper limit normal

## Abstract

**Background:**

Ammonia is implicated in hepatic encephalopathy (HE) and prognostic in cirrhosis. Venous ammonia concentration, yielding similar correlation with HE grades as arterial, has become the preferred practise but comparative data are limited.

**Aim:**

To quantify effect of sampling site on ammonia concentration in healthy persons and patients with cirrhosis.

**Methods:**

Ammonia concentrations were measured by arterial and femoral venous blood sampling in ten healthy men and ten male patients with cirrhosis before and during hyperammonaemia induced by ammonia infusion. Cubital vein samples were included during the infusion.

**Results:**

At baseline, arterial-venous concentration gaps were similar (*p* = 0.15) in healthy persons [14 (10–19) and 8 (4–12) µmol/L] and patients with cirrhosis [53 (32–74) and 40 (23–57) µmol/L]. Ammonia infusion increased arterial-venous concentration gaps in both groups [115 (97–133) and 61 (31–90) vs. 175 (123–227) and 134 (65–203) µmol/L]. Mean ammonia concentration difference between groups during hyperammonaemia was 72 (42–103) µmol/L (*p* < 0.001) and independent of sampling site. Cubital and femoral vein concentrations were comparable (*p* = 0.26). In cirrhosis, calculated upper limit normal values (ULN) were comparable for arterial and venous blood at baseline [2.0 (1.2–2.8) and 2.1 (1.2–3.0), *p* = 0.74] and during hyperammonaemia [6.7 (4.7–8.7) and 6.2 (4.4– 8.1), *p* = 0.44].

**Conclusions:**

We found clinically meaningful intra-individual arterial-venous concentration gaps in both healthy persons and patients with cirrhosis at any ammonia concentration. Inter-group concentration differences after induced hyperammonaemia were relatively constant across sampling sites which supports clinical use of venous sampling. ULN-normalised ammonia concentrations were valid for both arterial and venous sampling.

## Introduction

Ammonia, being neurotoxic, is implicated in the development of hepatic encephalopathy (HE). HE is a challenging diagnosis due to its nonspecific clinical features – and measurement of blood ammonia is especially relevant in ruling out HE owing to its high negative predictive value (European Association for the Study of the Liver 2022). Though elevated ammonia levels correlate with the severity of HE, the correlation is only moderate with substantial overlap across the various grades of HE (Ong et al. 2003; Nicolao et al. 2003). Moreover, ammonia measurements holds specific risk of errors related to blood collection on ice and requires rapid processing and analysis (Seligson and Hirahara 1957; Bajaj et al. 2020).

Therefore, the clinical utility of ammonia has been questioned (Bajaj et al. 2020; Haj and Rockey 2020; Tapper and Rahimi 2020). However, mounting evidence points to the prognostic potential of ammonia in liver disease with regard to risk of developing HE, decompensation and mortality in both in- and outpatients with liver cirrhosis (Vierling et al. 2016; Shalimar et al. 2019; Tranah et al. 2022; Balcar et al. 2023; Ballester et al. 2023). Thus, ammonia measurement is still relevant for both clinical and research purposes.

One aspect of the difficulties of ammonia measurements is the impact of blood sampling site. Inter-sample variation between arterial and venous sampling sites was studied by Stahl in the 1950s and 1960s (Stahl 1963). Based on these studies and from a theoretic point of view regarding arterial-delivered ammonia to the brain, arterial analysis was long considered gold standard (Stahl 1963). By arterial sampling, one also prevented falsely elevated ammonia levels related to the tourniquet of venous sampling. Since more recent studies examining correlations between ammonia levels and grades of HE showed similar results using arterial and venous sampling (Ong et al. 2003; Nicolao et al. 2003), eventually clinicians turned towards the easier obtainable venous sampling. Still, robust data regarding the effect of sampling site on ammonia concentration are scarce. Also, it remains unclear whether the possible effect of sampling site is comparable in healthy individuals and patients with cirrhosis and in conditions with low and high ammonia levels; not least given that cirrhosis associated sarcopenia could potentially decrease arterial-venous ammonia concentration gaps (Lattanzi et al. 2019).

Accordingly, the purpose of this study was to quantify the effect on ammonia levels of different sampling sites in healthy persons and patients with cirrhosis before and during induced hyperammonaemia. We, therefore, measured ammonia concentrations in arterial and femoral venous blood before and after infusion of ammonia as well as cubital veinous blood during the ammonia infusion.

## Materials and methods

### Study participants

Data obtained from a previous study using a constant ammonia infusion were available from ten healthy men and ten male patients with alcohol-related cirrhosis (Eriksen et al. 2023). The study included outpatients excluding patients with Child-Pugh score > 12, overt HE or more than one admission with HE within the past year. Informed consent was obtained from participants included in the study.

### Experimental set-up, blood sampling and ammonia analysis

At resting and fasting conditions, controlled hyperammonaemia was induced by a constant intravenous infusion of 0.25 mmol/kg/h ammonia chloride. At baseline and at steady state hyperammonaemia, 80 min after infusion start, blood was drawn synchronously from the radial artery and the femoral vein. In addition, cubital vein blood was drawn during hyperammonaemia, at t = 80 min.

Radial arterial and femoral venous blood was obtained by installed catheters and cubital venous blood by venepuncture with special care given to obtain blood stasis free after release of the tourniquet. By use of gas-tight adapters, after waste blood was drawn, blood was sampled and cooled directly on ice in di-potassium EDTA tubes. Plasma was immediately recovered after cooled centrifuging and stored at -40 °C until batch analysis the following day by enzymatic photometry (Siemens Healthcare, Erlangen, Germany; analytical variability for ammonia determination of 10–16% (2CV, twice the coefficient of variation). As previous described ammonia concentrations were adjusted for possible ammonia concentration drift due to plasma deamination processes after batch freezing and thawing (Eriksen et al. 2023).

### Statistics

Data were analysed using Stata version 14.2 (StataCorp, CollegeStation, TX) and presented as means with 95% CIs. Student’s paired and unpaired t test were used for comparisons within and between the groups, respectively. For skewed data, non-parametric Wilcoxon-Mann-Whitney test was used for comparisons between groups. The effects of sampling site and cirrhosis on ammonia concentration during induced hyperammonaemia were investigated using a multivariate linear mixed model. Equality of standard deviations and correlations in the model was considered as appropriate. Model validation was performed by comparing residuals and fitted values and by using QQ plots. The significance level was set at 0.05 in a two-tailed test. Normalised ammonia upper limit of normal (ULN) values for the respective sampling sites were calculated as the mean + 1.96 × SD of the measured value in healthy persons before ammonia infusion.

## Results

Basic characteristics of the study participants are presented in Table 1. The patients were older, had lower muscle mass and biochemical blood tests reflective of their liver disease and portal hypertension.

**Table 1:** Clinical and biochemical characteristics

	Healthy persons (*n* = 10)	Patients with cirrhosis (*n* = 10)	*p*
Age (years)	39 (22–66)	58 (43–73)	**< 0.001**
Body weight (kg)	85 (79–91)	77 (67–87)	0.17
Total muscle mass (kg)	31 (29–34)	26 (21–31)	**0.02**
Child-Pugh score^#^	-	7 (6–9)	-
MELD-Na score	-	12 (10–14)	-
Ascites (0/1/2/3)	-	8/0/2/0	-
*Biochemistry*			
Alanine aminotransferase (U/L)	20 (14–26)	33 (23–43)	**0.02**
Bilirubin (µmol/L)	12 (8–17)	23 (15–31)	**0.02**
Albumin (g/L)	40 (38–42)	28 (23–34)	**< 0.001**
Creatinine (µmol/L)	75 (67–84)	66 (55–77)	0.16
Haemoglobin (mmol/L)	8.5 (8.3–8.7)	7.1 (6.0–8.2)	**0.02**
Thrombocytes (x10^9^/L)	236 (195–277)	87 (58–116)	**< 0.001**
INR^#^	1.1 (1.0–1.1)	1.4 (1.2–2.2)	**0.003**

MELD-Na, model for end-stage liver disease-sodium; INR, international normalised ratio. Values are given as means with their 95% CIs.^#^Given as median (range)

Baseline arterial ammonia was lower in healthy persons as compared with the patients with cirrhosis (14 [10–19] µmol/L vs. 53 [32–74] µmol/L, *p* < 0.001, Table 2). Femoral vein concentrations were significantly lower in both groups (*p* ≤ 0.03) [8 (4–12) and 40 (23–57) µmol/L, respectively, < 0.001). The arterial-venous ammonia concentration gap did not differ significantly between the groups (*p* = 0.15). During ammonia infusion at t = 80 min, ammonia concentrations in the radial artery and the cubital and femoral veins were 115 (97–133), 61 (31–90), 47 (32–59) µmol/L, respectively, in healthy persons and 175 (123–227), 134 (65–203), 120 (84–155) µmol/L, respectively, in the patients with cirrhosis (Table 2).

**Table 2:** Ammonia concentrations before and after induced hyperammonaemia

	Healthy persons (*n* = 10)	Patients with cirrhosis (*n* = 10)	ULN values (for patients with cirrhosis)
Arterial, baseline	14 (10–19)	53 (32–74)	2.0 (1.2–2.8)
Femoral venous, baseline	8 (4–12)	40 (23–57)	2.1 (1.2–3.0)
Arterial, infusion	115 (97–133)	175 (123–227)	6.7 (4.7–8.7)
Femoral venous, infusion	47 (32–59)	120 (84–155)	6.2 (4.4– 8.1)
Cubital venous, infusion	61 (31–90)	134 (65–203)	-

Mean ammonia concentrations for healthy persons and patients with cirrhosis (µmol/L) and calculated upper limit normal (ULN) values for patients with cirrhosis with their respective 95% CIs

**Fig. 1:**
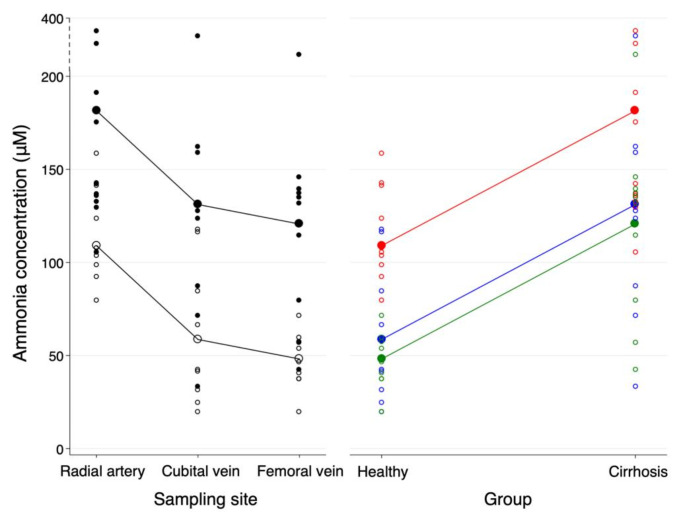
Interaction plots displaying the effect of sampling site (red: radial artery, blue: cubital vein, green: femoral vein) on ammonia concentration (μmol/L) during hyperammonaemia in healthy persons (white) and patients with cirrhosis (black). The additive multivariate model (larger circles) and the measured ammonia concentrations (smaller circles) are shown. Y-axis increments increases at y = 200. Mean ammonia concentration difference (μmol/L) between groups: 72 (42–103) (*p* < 0.001) and between sampling sites: a. radialis vs. v. cubitalis 50 (37–64) (*p* < 0.001), a. radialis vs. v. femoralis 61 (46–76) (*p* < 0.001), v. cubitalis vs. v. femoralis 10 (-8–29) (*p* = 0.26)

Data collected after 80 min of ammonia infusion were analysed by multivariate model testing showing no significant interaction between the effects of sampling site and cirrhosis (*p* = 0.75). Thus, the effect of sampling site were similar for healthy persons and cirrhosis patients during induced hyperammonaemia. Therefore, an additive model was chosen accepting parallel slopes between the two groups (Fig. 1). The average ammonia concentration difference between healthy persons and patients with cirrhosis across all three sampling sites was 72 (42–103) µmol/L *p* < 0.001. Table 3 presents ammonia concentration gaps between the radial artery, the cubital vein and the femoral vein. During induced hyperammonaemia, ammonia concentrations were non-significantly higher in the cubital vein as compared with the femoral vein (10 [-8–29] µmol/L, *p* = 0.26). In both groups, arterial concentrations were ~ 50 µmol/L (*p* < 0.001) higher than venous concentrations (Table 3).

**Table 3:** Ammonia concentration differences between radial artery, cubital vein and femoral vein during induced hyperammonaemia

Sampling site comparison	Δ [Ammonia]	*p*
a. radialis vs. v. cubitalis	50 (37–64)	**< 0.001**
a. radialis vs. v. femoralis	61 (46–76)	**< 0.001**
v. cubitalis vs. v. femoralis	10 (-8–29)	0.26

Mean ammonia concentration differences with their 95% CIs (Δ, µmol/L)

In the patients with cirrhosis, ammonia concentrations normalised to ULN yielded comparable values for arterial and venous blood at baseline [2.0 (1.2–2.8) and 2.1 (1.2–3.0) times ULN, respectively, *p* = 0.74] and during induced hyperammonaemia [6.7 (4.7–8.7) and 6.2 (4.4–8.1) times ULN, respectively, *p* = 0.44] (Table 2).

## Discussion

In this study we examined the impact of sampling site on ammonia concentrations before and after hyperammonaemia was induced by ammonia infusion. In both groups arterial ammonia was higher than femoral venous ammonia and this difference increased 3–4 fold during induced hyperammonaemia. Ammonia concentration differences between healthy individuals and patients with cirrhosis were comparable across the different sampling sites.

Higher arterial than venous ammonia concentrations are in accordance with a priori expectations as the resting skeletal muscle mass is a net ammonia-consuming organ (Huizenga et al. 1996; Lockwood 2004; Dam et al. 2011). Our observation on arteriovenous concentration gaps across the leg at basal conditions in healthy individuals align with reports from previous studies ranging from 5 to 14 µmol/L (Warter et al. 1983; Eriksson et al. 1985; Dam et al. 2011) as compared to 6 µmol/L in our study. As regards patients with cirrhosis, comparison between studies, not least between a controlled experimental set-up like ours and spontaneous ammonia concentration measurements from the clinic including patients with hepatic encephalopathy, is problematic. Nevertheless, similar relative arteriovenous concentration gaps for cirrhosis patients have been reported (Tyor et al. 1960; Stahl 1963; Ong et al. 2003; Nicolao et al. 2003; Dam et al. 2011; Manjunath et al. 2014); ranging from 10 to 35% as compared to 23% at baseline and 30% during hyperammonaemia in our study.

During induced hyperammonaemia we found clinically relevant differences between arterial and venous sampling sites of ~ 50 µmol/L for both healthy persons and cirrhosis patients, highlighting the sampling site for ammonia as being important when assessing hyperammonaemia. The small difference between ammonia concentration in cubital and femoral blood most likely reflect the fact that blood flow is proportional to muscle mass which thereby gives rise to similar fractional ammonia removal across the respective muscle group (arm vs. leg). Accordingly, depending on the clinical setting, different venous sampling sites may be used interchangeably for ammonia concentration measurement.

Of importance, during induced hyperammonaemia the ammonia concentration differences between healthy persons and patients with cirrhosis were tolerably constant across the three sampling sites in our analysis. Accordingly, discrimination between healthy persons and patients with cirrhosis was valid and quantitatively unaffected by venous sampling. The observation that arteriovenous differences during ammonia infusion were similar in healthy persons and patients with cirrhosis although the ammonia levels were higher in cirrhosis is interesting and needs further exploration that will also include measurement of muscle blood flow. We speculate that patients with cirrhosis have lower muscle mass and lower resting muscle blood flow resulting in reduced muscle ammonia removal but further explorations are needed.

Being a key pathological feature of liver disease, the primary driver of hepatic encephalopathy and prognostic in cirrhosis, we believe that ammonia measurement is still valuable even though the diagnostic specificity in hepatic encephalopathy is limited. Optimal and standardised sampling conditions and sampling site specific normal values is key. Our results indicates that the use of ULN values, as have been proposed (Tranah et al. 2022; Balcar et al. 2023; Ballester et al. 2023), is valid for both arterial and venous sampling sites, given that arterial- and venous- specific ULN values are used.

A major strength of our study is that we measured arterial and venous ammonia concentration gaps by synchronous sampling in each participant during standardised metabolic conditions during a controlled experimentally-induced hyperammonaemic steady state. Even though such hyperammonaemia is unphysiological, its impact on ammonia concentration in arterial versus venous sampling sites will likely mimic the clinical situation where systemic ammonia is increased in the patient with liver disease. Also, the careful sampling procedure limited preanalytical and analytical factors so our conclusions are not confounded this way. One limitation of our study is the small sample size that introduces the risk of type II error, e.g. regarding the importance of the numerically small effect of cubital vs. femoral sampling. Moreover, the study was not powered to explore the mechanistic explanation for the ammonia concentration differences observed between groups and sampling sites. Accordingly, a proper exploration of e.g., a possible correlation to liver dysfunction or muscle mass within the group of cirrhosis patients was not possible. We emphasise the importance of our healthy persons and controls being in the resting state; during physical exercise, ammonia production in the working muscle increases the ammonia concentration in venous blood draining these muscles and reduce arteriovenous concentration gaps (Eriksson et al. 1985). Thus, our results cannot be generalised to studies during exercise. Also, prolonged hyperammonaemia as observed in patients with hepatic encephalopathy due to e.g. a gastrointestinal bleed might exhaust muscle ammonia buffer capacity by depletion of carbon skeletons as glutamine is formed (from alpha-ketoglutarate and glutamate) which could potentially lower the arteriovenous concentration gaps.

In conclusion, in the basal state and during hyperammonaemia clinically important arterial-venous ammonia concentration gaps exist in both healthy persons and patients with cirrhosis. However, the concentration differences between healthy persons and patients with cirrhosis seems to be relatively constant across sampling sites, in support of the use of venous sampling for ammonia measurement in the clinical setting as long as it is clear what sampling site was used and interpretation takes sampling site into account. Also, ammonia concentrations normalised to calculated ULN values were valid for both arterial and venous sampling sites.

## Data Availability

No datasets were generated or analysed during the current study.
